# Supplementary material for: Direct growth of monolayer MoS2 on nanostructured silicon waveguides
Source: Nanophotonics. 2022 Aug 22;11(19):4397–408. doi: 10.1515/nanoph-2022-0235 (PMC11501977; doi:10.1515/nanoph-2022-0235)
Supplement: Supplementary file 1 — Supplementary Material Details [file j_nanoph-2022-0235_suppl.pdf]

Supplementary Information

Athira Kuppadakkath*, Emad Najafidehaghani*, Ziyang Gan, Alessandro Tuniz, Gia Quyet Ngo, Heiko Knopf, Franz J. F. Löchner, Fatemeh Abtahi, Tobias Bucher, Sai Shradha, Thomas Käsebier, Stefano Palomba, Nadja Felde, Pallabi Paul, Tobias Ullsperger, Sven Schröder, Adriana Szeghalmi, Thomas Pertsch, Isabelle Staude, Uwe Zeitner, Antony George, Andrey Turchanin*, and Falk Eilenberger*

Direct Growth of Monolayer MoS_2 on Nanostructured Silicon Waveguides

1 MoS_2 on S.O.I waveguide structure

1.1 Supplementary 1- Scanning-electron microscope (SEM) image

These SEM images compound Figure 2b of the manuscript and give detailed information about a set of TMD-coated waveguides. These images show the conformal growth of the 2D material.

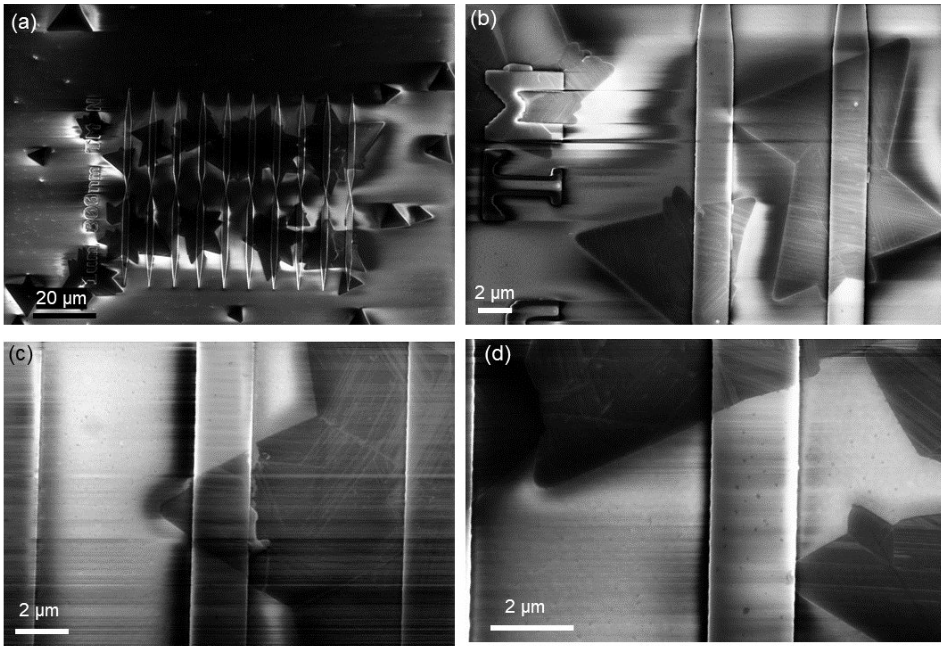

Fig. 1: Scanning electron microscopic (SEM) images of the MoS_2 crystals grown over the waveguide. a) SEM image of the waveguide structure with 1 μm long waveguide at the center and grating couplers (wide regions) at the top and bottom parts of the waveguide. b) Zoomed-in SEM image of the top part of the first two waveguide structures of Figure 1a. c) Zoomed-in SEM image of the top part of the third waveguide structure in Figure 1a. d) Zoomed-in image of the top part of the fourth waveguide structure in Figure 1a.

1.2 Supplementary 2- Atomic force microscope (AFM) measurements

The following AFM analysis is related to Figure 2c and Figure 2d of the manuscript. AFM height profiles are difficult to analyze because the substrate roughness exceeds the thickness of the monolayers. The crystal boundaries are nevertheless visible.

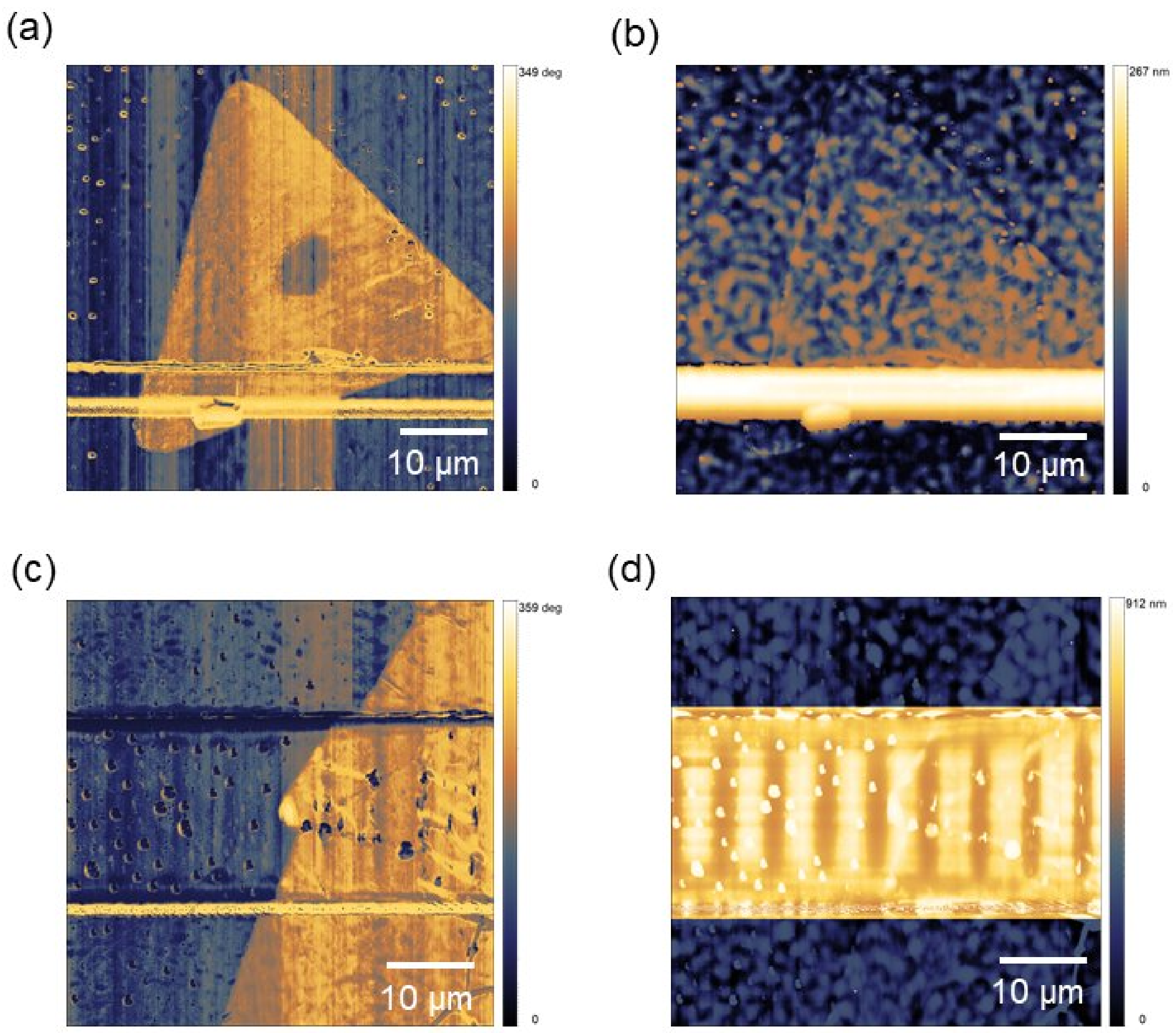

Fig. 2: a) AFM phase image of MoS_2 on the waveguide. b) AFM height map of the region in Figure 2a. c) AFM phase image of MoS_2 on the grating coupler. d) AFM height map of the region in Figure 2c.

1.3 Supplementary 3- Raman spectra measurements

The following is the discussion of the Raman map and spectra of the MoS_2 on the waveguide. An 18 cm^{-1} separation between the two peaks in the Raman spectra indicates the presence of monolayer MoS_2, whereas a separation of 22 cm^{-1} corresponds to multilayer. Most of the MoS_2 crystals grown on the structure are monolayers. The signal from Pos.2 in the Raman map was weak. So the integration time for this measurement is ten times larger than the measurements at the other two positions. The crystal at Pos.3 is a multilayer; this is also clear from the PL map in Figure 2a of the manuscript because there is no PL from

this area. The excitation wavelength was 530 nm. The microscope objective used for the excitation and collection had 100 × magnification.

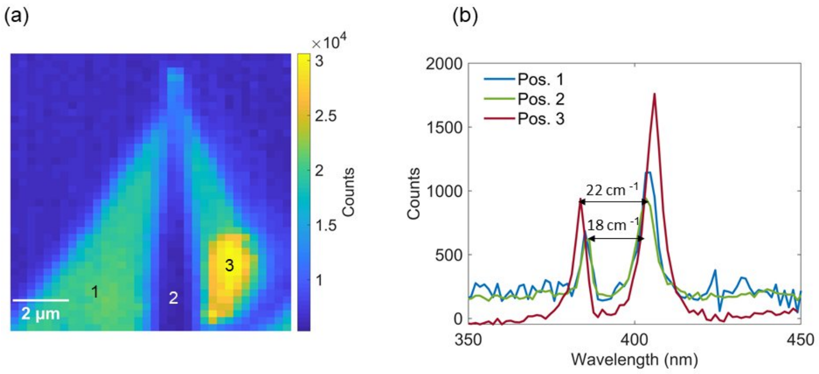

Fig. 3: a) Raman map. b) Raman spectra from the positions marked in the Raman map.

1.4 Supplementary 4- Functionality of waveguides

We checked whether the waveguide is functional after the high-temperature growth process of MoS_2. The waveguide was designed to operate at 1320 nm wavelength. We confirmed that the grating couplers, as well as the waveguide, are functional at the particular wavelength. In Figure 4b, we can see the waveguiding when the 1320 nm laser is excited on the grating couplers.

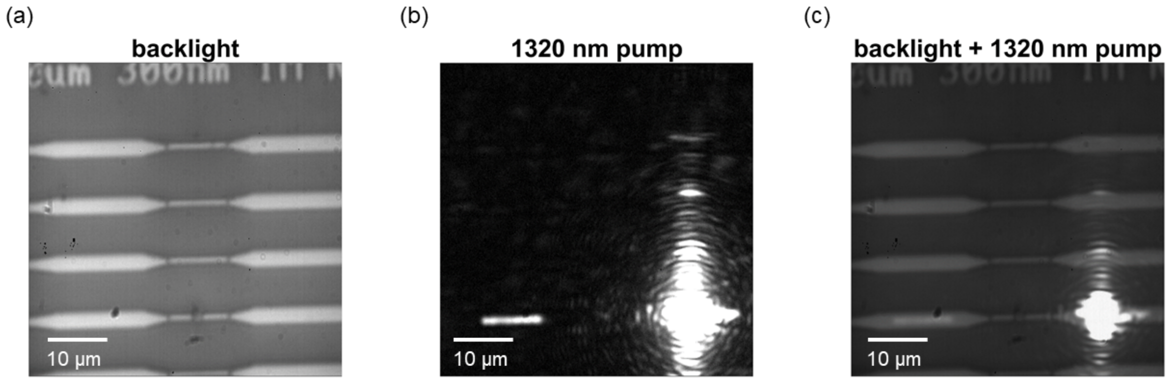

Fig. 4: Experiments showing waveguide functionality after high-temperature growth process. a) Waveguide structure in white light b) Waveguide excited with a laser emitting at 1320 nm wavelength. c) Waveguide excited with laser (1320 nm) in presence of white light.

1.5 Supplementary 5- Photoluminescence (PL) measurements

This supplement shows detailed PL measurement over a larger region discussed in Figure 3a of the manuscript. From these measurements, we see photoluminescence from the crystals showing the characteristic PL peak around 677 nm. The PL from the MoS_2 is reduced mainly on the grating couplers. The PL spectra from the crystals grown over the waveguide structure are similar to those grown on the plane substrate. This

shows that the emission properties of the crystals are not affected by the growth on the uneven surface. The PL signal intensity is reduced on places where MoS_2 is in contact with the Si waveguides. For this reason, the PL spectra d2, d4, and d7 are also weak at these positions.

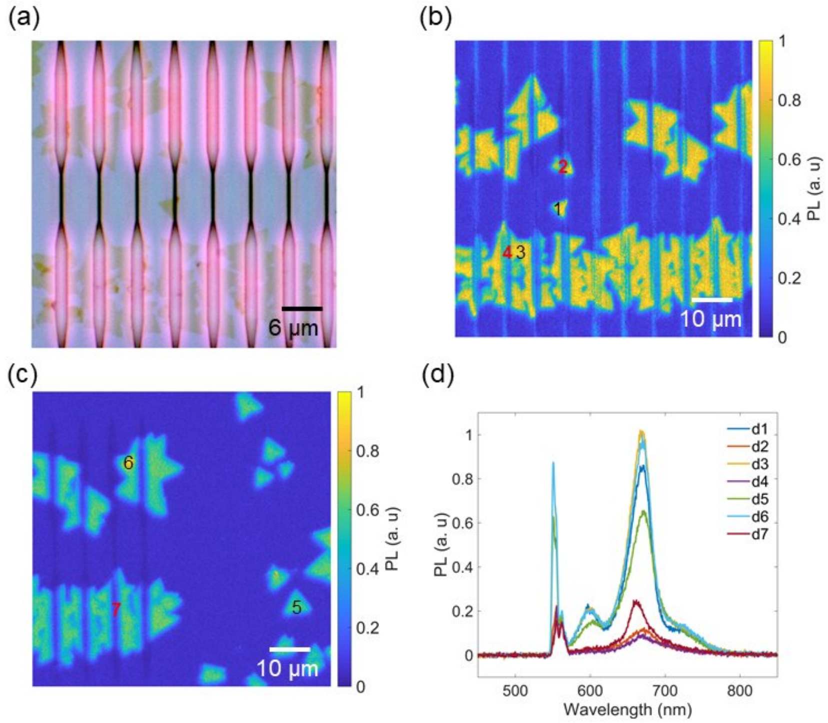

Fig. 5: a) Microscope image of the waveguide with the MoS_2 crystals. b) PL map of the region in Figure 5a. c) PL map of the region below the waveguide structure, where the crystals are grown on a plane surface. d) PL spectra from the marked positions in the PL maps.

1.6 Supplementary 6- Second harmonic (SH) measurements

SHG mapping is more sensitive toward modifications in the crystalline structure of the materials. The excitation was at 810 nm with the SHG at 405 nm. A double logarithmic plot of fundamental vs SHG power was fitted with a slope of 2.27, being in good agreement with the expected slope of 2.

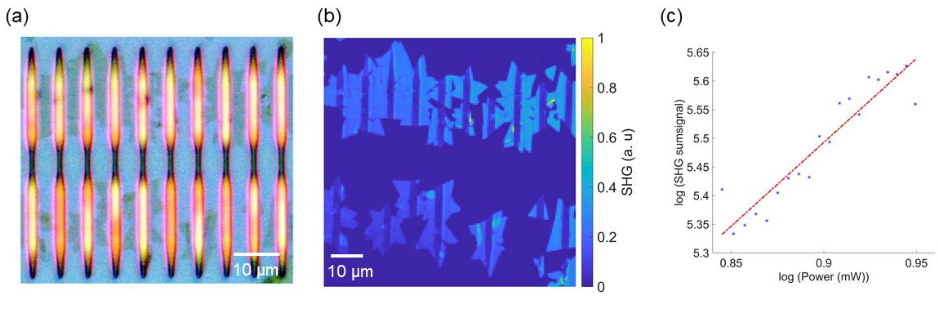

Fig. 6: a) Microscope image of a different waveguide structure on the sample. b) SHG map of the region shown in Figure 6a. c) Power dependency plot of Second harmonic signal.

2 MoS_2 on 5 nm Al_2O_3 coated S.O.I waveguide structure

As seen in Figure 5 and Figure 6 above, the emission properties (PL and SHG) of the MoS_2 are affected when it is in direct contact with Si. We hypothesize that a thin Al_2O_3 buffer layer between the Si and TMD would solve this issue without changing the waveguide properties considerably. Figure 7 is the plot of the effective index of the silicon waveguide as a function of the Al_2O_3 thickness 't' at a wavelength of 1.32 μm. For both the TE and TM modes, the change in the mode index is less than 1 % between the bare waveguide case and the 5 nm -coated case.

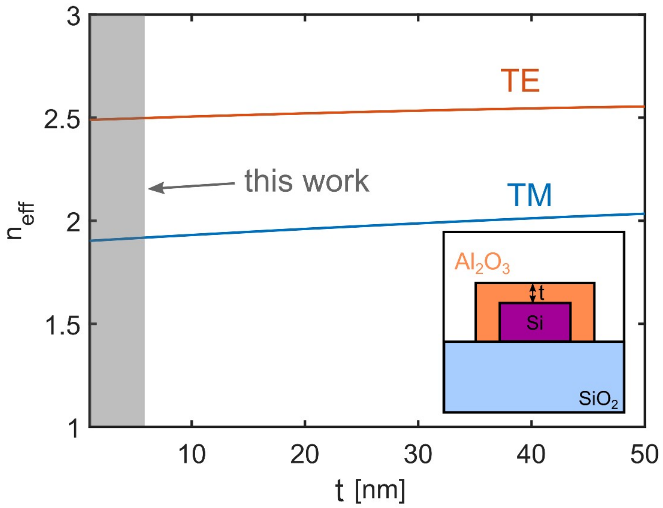

Fig. 7: Effective refractive indices of the TE and TM modes of the waveguide as a function of Al_2O_3 layer thickness. The refractive indices (n) used in the model are as follows. SiO_2: n= 1.45; Si: n=3.5; MoS_2: n = 4.2; air: n=1.

2.1 Supplementary 7- Photoluminescence (PL) measurements

More PL measurements (analogous to Figure 4b and Figure 4c of the manuscript) of the waveguide structures with 5 nm Al_2O_3 coating were carried out. The mapping was done to confirm our hypothesis that a buffer layer of 5 nm Al_2O_3 would prevent charge exchange between the TMD and the semiconducting waveguides and this prevents PL quenching.

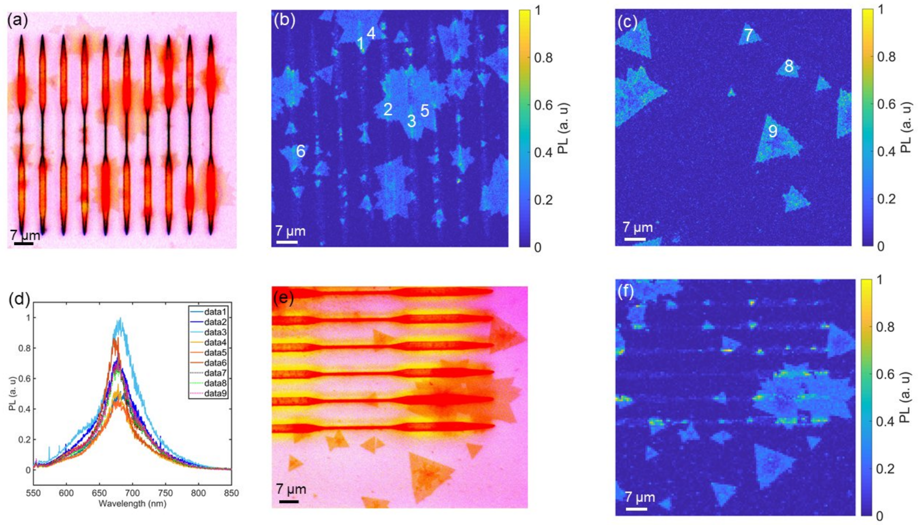

Fig. 8: a) Microscope image of MoS_2 grown on the waveguide structure with a dielectric layer of 5 nm Al_2O_3. b) PL map of the region shown in Figure 8a. c) PL map above the region of waveguide structure, where the crystals are grown on a plane surface. d) PL spectra at positions marked in the PL maps. e) Optical microscope image of a different waveguide structure on the sample. f) PL map of the region shown in Figure 8e.

2.2 Supplementary 8- Second harmonic (SH) measurements

The following figure is the SHG measurement of the same region discussed above in Figure 8a and Figure 8b. An area of $80\times$ 80 μm^2 is scanned to create the SHG map. Again we followed up on the hypothesis that the quenching of the excitons is prevented by the Al_2O_3 coating. Indeed, we find that the SHG intensity is no longer suppressed, to the contrary, it is enhanced from the waveguide regions. A comparison of the SHG map (Figure 9a) with the microscope image (Figure 8a) and PL map (Figure 8b) reveals an enhanced second harmonic signal from some of the multilayer regions present on top of the waveguide. Local field enhancement created by the increased density of states close to the high index waveguides could also enhance the SH signal on the grating structures. A detailed analysis will be the subject of future work.

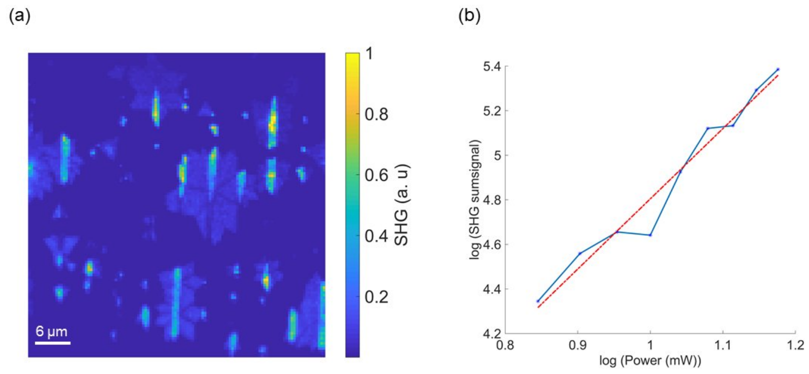

Fig. 9: a) Map of SH signal from the region discussed in Figure 7a and Figure 7b. b) Power dependency plot.

2.3 Supplementary 9- Raman spectra measurements

Raman measurement was performed to investigate the possible variation of strain fields as the TMDs grow over various substrates and around edges and corners. Following are additional Raman measurements related to Figure 4e in the manuscript. Strain effects are detected by the Raman shift of just the in-plane mode in the Raman spectra. Here we don't see an instance where just one of the Raman peaks is shifted. The spectra showing higher separation indicate the presence of multilayers on some parts of the waveguide. The excitation wavelength is 532nm.

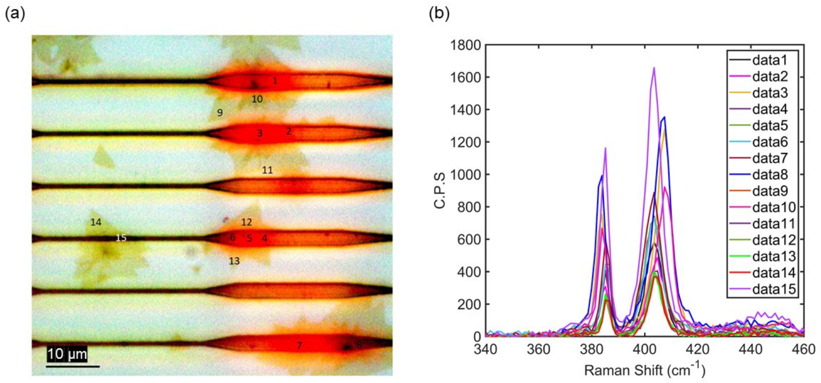

Fig. 10: a) Microscope image of the region of Raman measurement. b) Raman spectra measured from the positions marked in Figure 10a. The dark green regions of the MoS_2 crystal in the microscope image are identified as few layers because of the larger separation between the two peaks in those regions. In all other regions, the spectra look similar irrespective of whether they are on the waveguide structure part or on the surrounding SiO_2 part.

3 MoS_2 on 30 nm Al_2O_3 coated S.O.I waveguide structure

An additional waveguide sample was coated with 30 nm Al_2O_3 and then used for direct growth of MoS_2. We note that the Al_2O_3 protective coating has again performed its task: PL intensity is equally strong from the substrate as well as from the waveguides; Si-induced PL-quenching is thus suppressed. We even find some regions with slightly enhanced PL emanating directly from waveguides and some PL from regions, which are not coated by TMDs. This may hint at PL waveguiding but there were no occurrences of the phenomenon to warrant systematic investigation.

3.1 Supplementary 10- Photoluminescence (PL) and Second-harmonic measurements

The following Figure is the optical characterization of a different region of the sample coated with 30 nm Al_2O_3. In this case, we see a less second harmonic signal from the multilayer part of the MoS_2 crystal, which looks like dots at the center of the crystal in the optical microscope image.

3.2 Supplementary 11- AFM before and after CVD process

We performed AFM measurements on the waveguide grating couplers before and after the CVD growth process to ensure that the high temperature required for the growth of the 2D material didn't damage the grating couplers substantially.

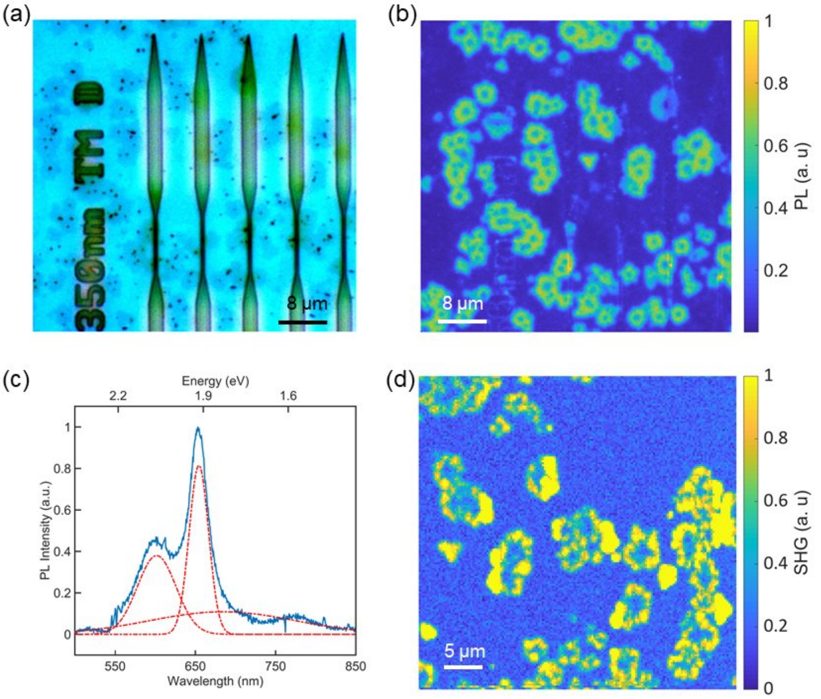

Fig. 11: a) Microscope image of a section of the Si-chip which contains five grating couplers and waveguides coated with MoS_2 monolayer crystal. b) PL map of MoS_2 monolayers on silicon waveguide structure. c) PL spectra from the MoS_2 crystal (blue spectra) and the experimental data fitted with a double Gaussian fit (dashed red curve). d) Second harmonic map of the MoS_2 coated waveguide structure.

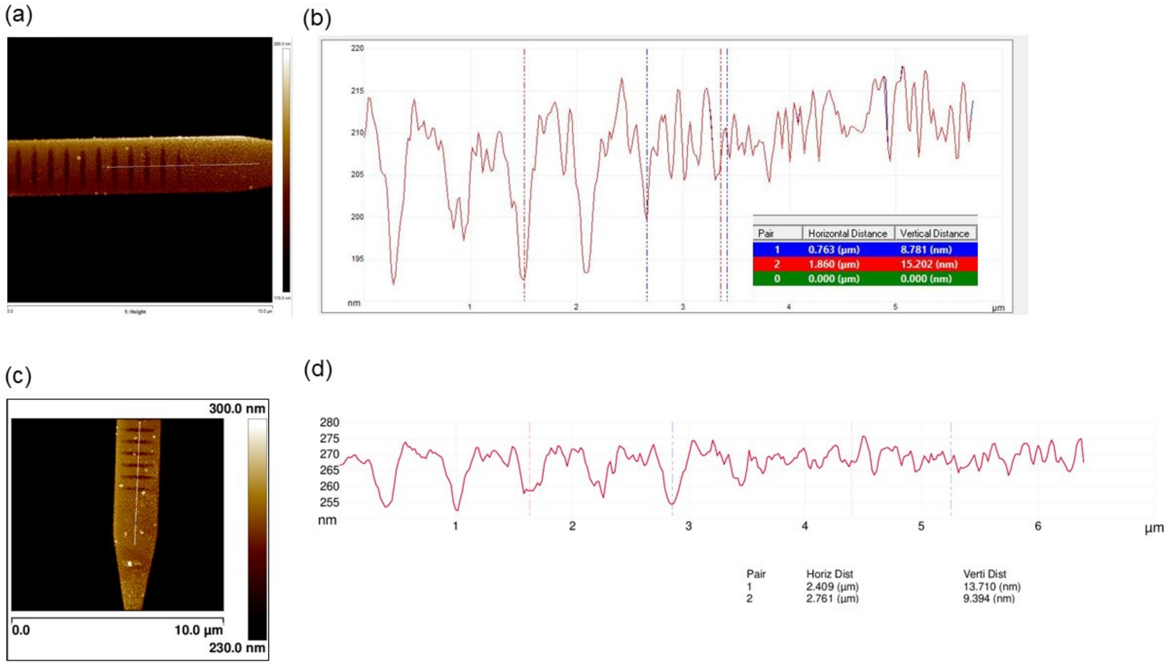

Fig. 12: a) AFM height map before the CVD process. b) AFM height profile across the line in Figure 12a. c) AFM height map after the CVD process. d) AFM height profile across the gratings.
